# PKM2 is the target of proanthocyanidin B2 during the inhibition of hepatocellular carcinoma

**DOI:** 10.1186/s13046-019-1194-z

**Published:** 2019-05-17

**Authors:** Jiao Feng, Liwei Wu, Jie Ji, Kan Chen, Qiang Yu, Jie Zhang, Jiaojiao Chen, Yuqing Mao, Fan Wang, Weiqi Dai, Ling Xu, Jianye Wu, Chuanyong Guo

**Affiliations:** 1 Department of Gastroenterology, Shanghai Tenth People’s Hospital, Tongji University School of Medicine, NO. 301, Middle Yanchang Road, Jing’an District, Shanghai, 200072 China; 2 Shanghai Tenth Hospital, School of Clinical Medicine of Nanjing Medical University, Shanghai, 200072 China; 3 Department of Gerontology, Shanghai General Hospital, Shanghai Jiaotong University School of Medicine, Shanghai, 200080 China; 4 Department of Oncology, Shanghai General Hospital, Shanghai Jiaotong University School of Medicine, Shanghai, 200080 China; 5 Department of Gastroenterology, Zhongshan Hospital of Fudan University, Shanghai, 200032 China; 6 Shanghai Institute of Liver Diseases, Zhongshan Hospital of Fudan University, Shanghai, 200032 China; 7 Department of Gastroenterology, Shanghai Tongren Hospital, Shanghai Jiaotong University School of Medicine, Shanghai, 200336 China; 8 Department of Gastroenterology, Putuo People’s Hospital, Tongji University School of Medicine, NO. 1291, Jiangning Road, Putuo District, Shanghai, 200060 China

**Keywords:** Proanthocyanidin B2, Hepatocellular carcinoma, Aerobic glycolysis, PKM2, Sorafenib

## Abstract

**Background:**

The treatment for advanced primary hepatocellular carcinoma (HCC) is sorafenib (SORA), while HCC has become increasingly drug resistant with enhanced aerobic glycolysis. The present study aimed to examine the chemotherapeutic effects of a flavonoid proanthocyanidin B2 (PB2) on HCC.

**Methods:**

Five kinds of HCC cell lines and LO2 were used to test the effect of PB2 on aerobic glycolysis. The proliferation, cell cycle, apoptosis and a xenograft mouse model were analyzed. Lentivirus overexpressed pyruvate kinase M2 (PKM2) or sh-PKM2 was used to verify the target of PB2. The detailed mechanism was investigated by immunofluorescence, co-immunoprecipitation, and western blotting.

**Results:**

PB2 inhibited the proliferation, induced cell cycle arrest, and triggered apoptosis of HCC cells in vivo and in vitro. PB2 also suppressed glucose uptake and lactate levels via the direct inhibition of the key glycolytic enzyme, PKM2. In addition, PKM2 inhibited the nuclear translocation of PKM2 and co-localization of PKM2/HIF-1α in the nucleus, leading to the inhibition of aerobic glycolysis. Co-treatment with PB2 was also effective in enhancing the chemosensitivity of SORA.

**Conclusions:**

PB2 inhibited the expression and nuclear translocation of PKM2, therefore disrupting the interaction between PKM2/HSP90/HIF-1α, to suppress aerobic glycolysis and proliferation, and trigger apoptosis in HCC via HIF-1α-mediated transcription suppression.

**Electronic supplementary material:**

The online version of this article (10.1186/s13046-019-1194-z) contains supplementary material, which is available to authorized users.

## Background

Primary liver cancer includes hepatocellular carcinoma (HCC) (75–85%), intrahepatic cholangiocarcinoma (10–15%), and some other rare types of HCC [1]. According to the latest reports, liver cancer has become the sixth most commonly diagnosed cancer and the fourth leading cause of cancer death worldwide in 2018 [2]. China is one of the most high risk areas of HCC because of the high incidence of chronic hepatitis B/C viruses (HBV/HCV) infection [3]. Therefore, the treatment of HCC is important for the control of HCC mortality.

Except for HBV/HCV infection, aflatoxin, tobacco use, alcohol abuse, nonalcoholic fatty liver disease, diabetes, and obesity are also risk factors of HCC [4, 5]. The pathogenesis of HCC is complex, which includes the suppression of apoptosis, regulation of the cell cycle, the activation of oncogenes, and the inhibition of tumor suppressor genes [6, 7]. Otto Warburg observed that even in the presence of oxygen, cancer cells undergo a shift in energy production from oxidative phosphorylation (OXPHOS) to glycolysis, which was termed the “Warburg effect” or “aerobic glycolysis” [8, 9]. Aerobic glycolysis is also present in HCC, and targeting aerobic glycolysis has become a new chemotherapeutic strategy for the treatment of HCC [9–11].

Aerobic glycolysis is the process by which glucose is transferred to lactate in the cytoplasm, instead of carbon dioxide in the mitochondria. There are three rate-limiting enzymes during glycolysis, including hexokinase (HK), fructose-6-phosphokinase (PFK), and pyruvate kinase (PK) [12]. Among them, PK is the most important because it controls the final conversion of phosphoenolpyruvate to pyruvate [13]. There are four isoforms of PK (L, R, M1, and M2), and PKM2 has been found to be dramatically increased in liver cancer cells, and plays a critical role in the regulation of glycolysis [14]. Furthermore, studies have reported that targeting PKM2 enhances the therapeutic effect of cancer, including HCC [15, 16].

Proanthocyanidin B2 (PB2) is a type of dimer flavonoid that is found in grape seed, pine bark, wine, and tea leaves [17]. PB2 has been shown to possess various bioactivities, including anti-oxidant, anti-inflammation, and anti-obesity activities, and it has also shown efficacy in the treatment of cancer, cardiovascular disease, type 2 diabetes, ulcerative colitis, as well as acute liver injury [17–20]. Therefore, PB2 has been considered as a bioactive food component, and PBs-rich functional foods are commercially available. However, there have been a limited number of studies describing the effect of PB2 on HCC and its metabolism.

Surgical resection and liver transplantation are currently the only possible curative treatment options for HCC [6]. It is therefore important to develop novel agents for HCC treatment. To provide the basis for the development of more effective treatments, the present study investigated the effect of PB2 on HCC cells and HCC metabolism, especially during aerobic glycolysis.

## Materials and methods

### Reagents

PB2, pentobarbital, dimethyl sulfoxide (DMSO), puromycin and polybrene were purchased from Sigma-Aldrich (St. Louis, MO, USA). Sorafenib tosylate (SORA), DASA 58, FG-4592 and BAY87–2243 were purchased from Selleck Chemicals (Shanghai, China). The Cell Counting Kit (CCK-8) and the Hoechst 33258 fluorescence staining kit were obtained from Yeasen Biotechnology (Shanghai, China). The annexin V-FITC apoptosis detection kit and PI/RNase Staining Buffer were purchased from BD Biosciences (San Jose, CA, USA). Penicillin and streptomycin were purchased from Gibco (Thermo Fisher Scientific, Waltham, MA, USA) and high glucose Dulbecco’s Modified Eagle Medium (DMEM) and fetal bovine serum (FBS) were purchased from HyClone (GE Healthcare, Logan, UT, USA). Oligonucleotide primers were synthesized by Generay Biotech (Shanghai, China). The PrimeScript RT Reagent kit and SYBR Premix Ex Taq were purchased from TaKaRa Biotechnology (Dalian, China). Detailed information of the primary antibodies used in this study are listed in Table 1. Anti-goat or anti-mouse secondary antibodies were obtained from Dako (Santa Clara, CA, USA).

**Table 1:** The primary antibodies used in the study

Antibody	Species	Targeted species	Dilution ratio	Supplier	Catalogue number
β-actin	M	H, M, R	1:1000	CST	3700
PCNA	Rbt	H, M, R	1:2000	PT	10,205–2-AP
Bax	M	H, M, R	1:1000	PT	60,267–1-Ig
Caspase 3	Rbt	H, M, R	1:1000	PT	19,677–1-AP
Caspase 9	M	H, M, R	1:1000	PT	66,169–1-Ig
HK2	Rbt	H, M, R	1:1000	PT	22,029–1-AP
PFKFB3	Rbt	H, M, R	1:1000	CST	13,123
PKM2	M	H, M, R	1:1000	PT	60,268–1-Ig
OXPHOS	M	H, M, R	1:250	MT	MS604
HIF-1α	Rbt	H	1:1000	PT	20,960–1-AP
HSP90	M	H, M, R	1:1000	PT	60,318–1-Ig
LaminA/C	R	H, M, R	1:1000	PT	10,298–1-AP
NF-κB	R	H, M, R	1:1000	PT	10,745–1-AP
Stat3	M	H, M, R	1:1000	CST	9139
p-Stat3(Tyr705)	R	H, M, R	1:1000	CST	9145
GLUT1	R	H, M, R	1:100	PT	21,829–1-AP

Abbreviations for the table: H, human; M, mouse; Rbt, rabbit; R, rat; CST, Cell Signaling Technology (Danvers, MA, USA). PT, Proteintech (Chicago, IL, USA). Abcam (Cambridge, MA, USA). MS, Mitoscience (St. Louis Park, MN, USA)

### Cell lines and cell culture

The LO2 normal human liver cell line and the HCC-LM3, SMMC-7721, Bel-7402, Huh-7 and HepG2 human hepatocarcinoma cell lines were obtained from the Cell Bank of Type Culture Collection of the Chinese Academy of Sciences (Shanghai, China), and were cultured in high glucose DMEM supplemented with 10% FBS, 100 U/mL of penicillin, and 100 g/mL of streptomycin in a humidified incubator at 37 °C with 5% CO_2_. All experiments were performed in triplicate.

### CCK8 assay

Apparent logarithmic phase cells were seeded in 96-well plates at a density of 2 × 10^4^/mL for 24 h, then PB2 was added at concentrations of 10, 20, 40, 60, 80, 100, 120, or 140 μM for 24, 48, or 72 h. Cell viability was then measured using the CCK8 assay according to the manufacturer’s protocol. Cell viability was expressed as a percentage of control cells.

### Colony formation

Cells were seeded at 2000 cells/well in 6-well plates for 6 days. On day 7, 60, 80, and 100 μM PB2 was added to treat the cells for another 6 days. At the end of treatment, the cells were fixed with 95% ethanol and stained with 0.1% Crystal Violet. The number of colonies formed was then counted.

### Flow cytometry analysis of the cell cycle

Cells were seeded in 12-well plates for 24 h, and treated with 60, 80, or 100 μM PB2 for 24 h. The cells were then harvested and fixed with 75% ethanol. The distribution of the cell cycle was determined using propidium iodide (PI) staining and flow cytometry (FACSCalibur; Becton, Dickinson, Franklin Lakes, NJ, USA) analysis according to the manufacturer’s protocol. The results were analyzed with ModFit LT software (Verity Software House, Topsham, ME, USA).

### Flow cytometry analysis of apoptosis

Cells were seeded in 12-well plates for 24 h, and treated with 80 μM PB2 for 24 h. Cells were then harvested and stained with annexin V-FITC and PI, and detected by flow cytometry. The results were analyzed by FlowJo software (version 10; FlowJo LLC, Ashland, OR, USA). The annexin V-positive and PI-negative cells were regarded as early apoptosis cells, and annexin V-positive and PI-positive cells were regarded as late apoptosis/secondary necrosis cells.

### Hoechst 33258 staining

Cells were seeded in 6-well plates for 24 h, and treated with 80 μM PB2 for 24 h. Cells were then washed with phosphate-buffered saline (PBS) for three times and fixed with 75% ethanol for 10 min. The morphological changes of apoptosis were detected using the Hoechst 33258 fluorescence staining kit according to the manufacturer’s protocol. The apoptotic cells were characterized by bright blue fluorescence in the nucleus.

### Biochemical assays

The glucose uptake level was determined via the uptake of 2-[^3^H] deoxyglucose by cells as previously described, and the values were normalized to the protein concentrations of the cell lysates [11, 21]. The lactate concentration of the cell supernatant or the tumor tissues were determined using a Lactate Testing Kit (Nanjing Jiancheng Bioengineering Institute, Nanjing, China), according to the manufacture’s protocol.

### Reverse transcription-polymerase chain reaction (RT-PCR) and quantitative real-time PCR (qPCR)

The total RNA of cells was extracted using the TRIzol agent, and the mRNA was reversed transcribed into cDNA by RT-PCR following the manufacturer’s protocol. The primers used for qPCR are listed in Table 2. The protocol used was: 95 °C × 3 min, 95 °C × 3 s, 60 °C × 30 s × 40 cycles, which was performed using a 7900HT Fast PCR System (Applied Biosystems, Foster City, CA, USA). The relative fold induction was quantified using the 2^-ΔΔCt^ method [22].

**Table 2:** Primers used for qPCR

Gene name	Forward (5′-3′)	Reverse (5′-3′)
HSP90AA1	AGGAGGTTGAGACGTTCGC	AGAGTTCGATCTTGTTTGTTCGG
HSP90AB1	AGAAATTGCCCAACTCATGTCC	ATCAACTCCCGAAGGAAAATCTC
PKM2	ATGTCGAAGCCCCATAGTGAA	TGGGTGGTGAATCAATGTCCA
HK2	GAGCCACCACTCACCCTACT	CCAGGCATTCGGCAATGTG
PFKFB1	AGAAGGGGCTCATCCATACCC	CTCTCGTCGATACTGGCCTAA
PFKFB2	TGGGCCTCCTACATGACCAA	CAGTTGAGGTAGCGTGTTAGTTT
PFKFB3	TTGGCGTCCCCACAAAAGT	AGTTGTAGGAGCTGTACTGCTT
PFKFB4	TCCCCACGGGAATTGACAC	GGGCACACCAATCCAGTTCA
LDH-A	ATGGCAACTCTAAAGGATCAGC	CCAACCCCAACAACTGTAATCT
LDH-B	TGGTATGGCGTGTGCTATCAG	TTGGCGGTCACAGAATAATCTTT
LDH-C	AGAACATGGTGATTCTAGTGTGC	ACAGTCCAATAGCCCAAGAGG
HIF-1α	GAACGTCGAAAAGAAAAGTCTCG	CCTTATCAAGATGCGAACTCACA
AMPK-α1	TTGAAACCTGAAAATGTCCTGCT	GGTGAGCCACAACTTGTTCTT
AMPK-α2	GTGAAGATCGGACACTACGTG	CTGCCACTTTATGGCCTGTTA
AMPK-β1	CCACTCCGAGGAAATCAAGGC	CTGGGCGGGAGCTTTATCA
GLUT1	GGCCAAGAGTGTGCTAAAGAA	ACAGCGTTGATGCCAGACAG
β-actin	CATGTACGTTGCTATCCAGGC	CTCCTTAATGTCACGCACGAT

### Western blotting analysis

Total protein was extracted from cells using RIPA lysis buffer. The nuclear and cytoplasmic protein was extracted using a commercial kit (Nuclear and Cytoplasmic Protein Extraction Kit, Yeasen Biotechnology, Shanghai, China). The protein concentration was determined with a BCA kit, and 30 μg of protein was loaded and then transferred to a polyvinylidene fluoride membrane (Millipore, Billerica, MA, USA). After blocking with 5% nonfat milk for 1 h at room temperature, the membranes were incubated with the primary antibodies at 4 °C overnight. The next day, after three washings in PBST (1% Tween diluted in PBS), the membranes were incubated with secondary antibodies at room temperature for 1 h. The membranes were finally scanned with an Odyssey (Licor, Lincoln, NE, USA).

### Immunofluorescence (IF) and double IF staining

Cells were seeded in glass coverslips for 24 h, and treated with 80 μM PB2 for 24 h. The treated cell slices were fixed with 4% paraformaldehyde, washed with PBS, and incubated with primary antibodies against PKM2, hypoxia-inducible factor (HIF)-1α, or heat shock protein 90 (HSP90) at 4 °C overnight. The next day, after incubation with fluorescence secondary antibody and 4′,6-diamidine-2-phenylindole, the slices were mounted and observed with an inverted fluorescence microscope (Leica DMIRB, Buffalo Grove, IL, USA). All double IF images were captured under the same conditions. The co-localization analysis was performed using Image J software (NIH Image, Bethesda, MD, USA).

### Plasmid construction, lentivirus packaging, and cell transfection

The PKM2 overexpression or knockdown lentivirus was synthesized by BioLink Biotechnology (Shanghai, China). Briefly, to create a recombinant plasmid overexpressing PKM2 (PKM2-OE), a full-length cDNA encoding the PKM2 sequence was amplified and cloned into the pLenO-GTP vector. To generate a recombinant plasmid expressing PKM2-shRNA (sh-PKM2), double-stranded oligonucleotides were cloned into the pLenR-GPH vector. Both plasmid sequences were verified by DNA sequencing. Empty vector (EV) was used as a control. All recombinant lentiviruses were then generated from HEK-293 T cells using calcium phosphate precipitation.

To establish a stable lentivirus transfection HCC cell line, HCC-LM3 and SMMC-7721 cells were seeded in 6-well plates, and when 60–70% confluent, they were transfected with EV, PKM2, or sh-PKM2 lentivirus in the presence of 5 μg/mL polybrene for 24 h. The positive cells were selected by puromycin and transfection efficiency was determined by qPCR and western blotting.

### Co-immunoprecipitation (co-IP) assay

Cells were seeded in 10 cm dishes, and then treated with 80 μM PB2 for 24 h. The cells were lysed at 4 °C in ice-cold IP Lysis/Wash Buffer containing protease inhibitors. The Co-IP assay was then performed using the Pierce Co-immunoprecipitation Kit (Thermo Scientific, Waltham, MA, USA) according to the manufacture’s protocol.

### Animals and establishment of xenograft tumor model

Five-week-old male BALB/C nude mice were obtained from Shanghai SLAC Laboratory Animal (Shanghai, China) and housed in a standard animal laboratory with free access to food and water. The apparent logarithmic phase HCC-LM3 cells were suspended in serum-free DMEM (3 × 10^6^/mL) and injected into the upper flank region of mice (200 μL each). The tumor volume was calculated using the following formula: volume (mm^3^) = 0.5 × (major axis) × (minor axis)^2^. When the xenograft tumor volume reached 100 mm^3^, the animals were randomly divided into the following groups: for the analysis of the PB2 effect, 10 mice were randomly divided into two groups (*n* = 5): (1) for the normal control (NC) group, mice were treated with normal saline only; and (2) for PB2 group, mice were treated with 100 mg/kg PB2by gavage once a day for 30 days [18, 23, 24]. For the combination treatment with SORA, 16 mice were randomly divided into four groups (*n* = 4): (1) NC group; (2) PB2 group; (3) for the SORA group, mice were treated with 10 mg/kg SORA by gavage once a day for 30 days; [10] and [4] for the PB2 + SORA group, mice were treated with both 100 mg/kg PB2 and 10 mg/kg SORA by gavage once a day for 30 days.

To verify by targeting on PKM2, the effect of PB2 on HCC in vivo, another nine mice were used. They were divided into three groups (*n* = 3): (1) EV group; (2) PKM2-OE group; (3) PKM2-OE + PB2 group, which were injected with stable transfected EV LM3 or PKM2-OE LM3 cells in the upper flank region. When the tumor volume in PKM2-OE + PB2 group reached 100 mm^3^, the mice were treated with 100 mg/kg PB2 by gavage once a day for 30 days.

At the end of the experiments, mice were anesthetized with 1.25% pentobarbital (40 mg/kg, intraperitoneally), and the tumors were resected and imaged, then immersed in 4% paraformaldehyde. The heart, kidney, and lung of the mice in the NC and PB2 groups were also separated for toxicity analyses.

### Hematoxylin and eosin (H&E) staining, immunohistochemistry (IHC), and terminal deoxynucleotidyl transferase dUTP nick end labeling (TUNEL) assay

Paraformaldehyde-immersed tissues were embedded in paraffin and cut into 3 μm thick sections. For H&E staining, the slices were stained with hematoxylin for 10 min and eosin for 5 min to visualize tissue injuries. For IHC staining, the slices were dewaxed and rehydrated, and after an antigen retrieval process and blocking, they were incubated with primary antibodies against PKM2 or GLUT1 overnight. For the TUNEL assay, the slices were dewaxed, dehydrated, and then digested with 20 μg/mL proteinase K at room temperature for 15 min. The slices were then incubated in the TUNEL reaction mixture at room temperature for 1 h. The brown-stained cells were regarded as TUNEL-positive cells. All of the images were captured by an optical microscope.

### Statistical analysis

Each experiment was repeated at least three times. All quantitative data are expressed as the mean ± standard deviation (SD) and imaged using GraphPad Prism 6 software (GraphPad Software, San Diego, CA, USA). The comparisons between two groups were analyzed by Student’s *t*-tests (unpaired, two-tailed) or the one-way analysis of variance (ANOVA) (followed by Tukey’s post-hoc tests). Statistical significance was defined as *P* < 0.05.

## Results

### PB2 inhibited the proliferation, and induced cell cycle arrest and apoptosis of HCC cells

**Fig. 1:**
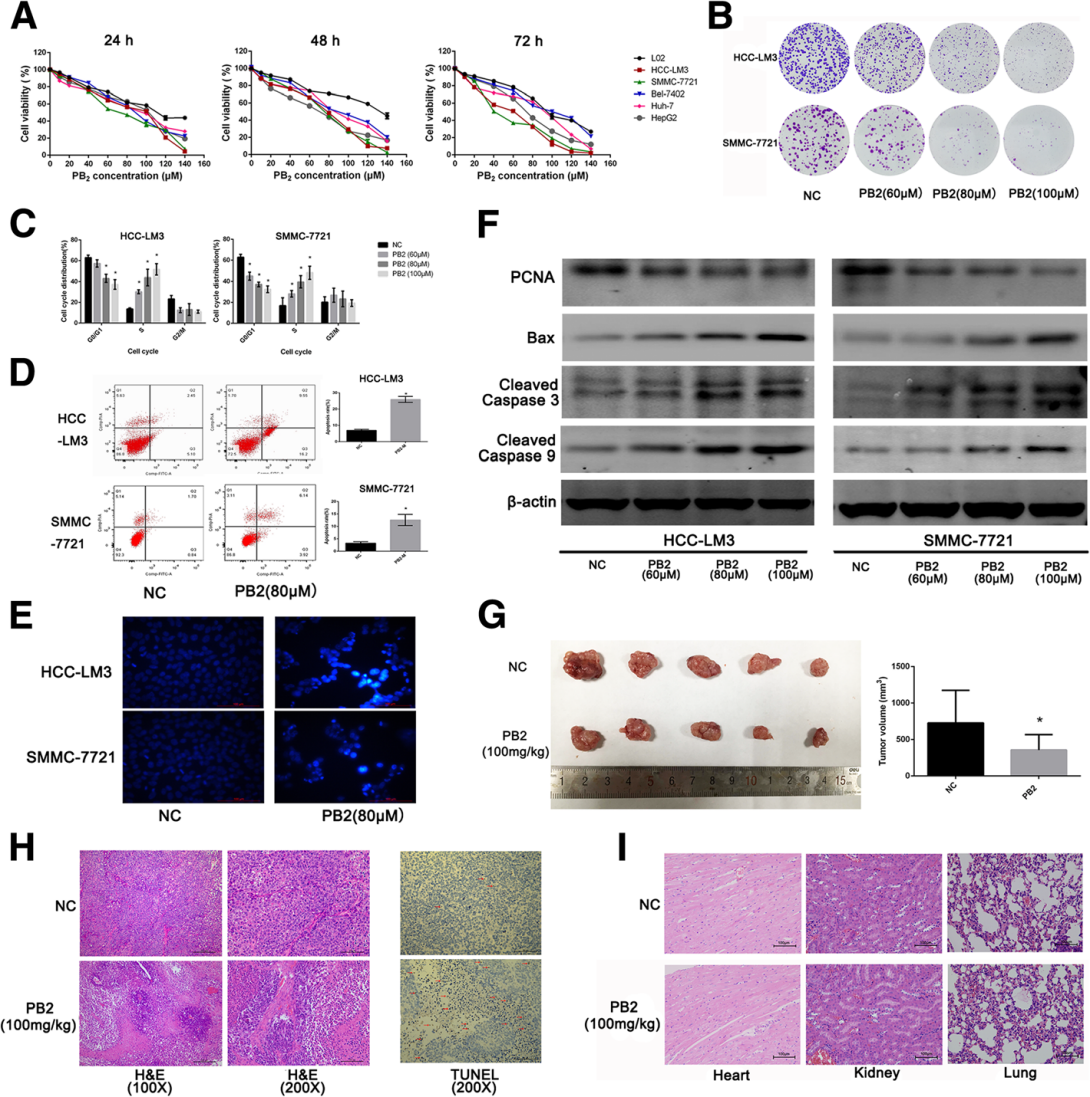
The effects of PB2 on the proliferation, cell cycle, and apoptosis of HCC in vitro and in vivo. **a** The cell viability was determined using the CCK8 assay after PB2 treatment for 24, 48, and 72 h. **b** The colony formation of HCC-LM3 and SMMC-7721 cells. **c** Cell cycle distribution. PB2 caused cell cycle arrest in the S phase. **d** Flow cytometry analysis of apoptosis. **e** Hoechst 33328 staining of HCC cells (original magnification, 200×). The apoptotic cells were stained with bright blue fragments and fluorescence in the nucleus. **f** Western blot analysis of proliferation- and apoptosis-related markers. **g** The gross manifestation and volumes of tumors (*n* = 5). **h** The hematoxylin and eosin (H&E) staining and TUNEL staining of tumor sections in both NC and PB2 groups (original magnification, 100× or 200×). There were many cancer cells and neo-vessels diffused in the solid neoplastic tissue in the NC group, while there were more lesions of necrosis and less neo-vessels in the PB2 group. In TUNEL staining, apoptotic cells were indicated by red arrows. **i** The H&E staining of heart, kidney, and lung from the NC and PB2 groups (original magnification, 200×). PB2 treatment for a month did not harm these organs. *P < 0.05 vs. the NC group (1**c**: one-way ANOVA; 1**d**&1**d**: Student’s t-test)

The CCK8 assay was used to determine the toxicity of PB2 on HCC and normal liver cell lines. As shown in Fig. 1a, PB2 inhibited the viability of five kinds of HCC cell lines in a dose- and time-dependent manner. Low doses of PB2 did not affect the viability of the LO2 normal liver cell line, suggesting that PB2 could inhibit the proliferation of cancer cells without affecting normal cells. HCC-LM3 and SMMC-7721 cells were very sensitive to PB2, with a half maximal inhibitory concentration (IC_50_) of 80.59 μM and 76.32 μM at 24 h, respectively. To further determine the effects of PB2 on the proliferation of HCC-LM3 and SMMC-7721 cells, the colony formation results showed that PB2 inhibited the proliferation and colony formation ability of these cells (Fig. 1b). The protein expression levels of proliferating cell nuclear antigen (PCNA), by western blotting, confirmed that PB2 inhibited proliferation (Fig. 1f).

The inhibition of cell proliferation is accompanied by cell cycle and apoptosis regulation [6]. Our results showed that PB2 treatment also increased the proportion of HCC cells in the S phase and decreased the proportion in the G0/G1 phase, indicating that PB2 caused cell cycle arrest in S phase (Fig. 1c). The flow cytometry analysis showed that 80 μM PB2 induced HCC cell apoptosis, which was reflected by an increase in the apoptosis rate. The Hoechst 33328 stain also showed that there were more nuclear fragments and bright blue fluorescence in PB2-treated cells than in the NC groups, indicating morphological changes due to apoptosis (Fig. 1d). Western blot analyses of Bax, cleaved caspase 3, and cleaved caspase 9, which were all apoptosis related proteins, showed that PB2 increased the expression of these biomarkers (Fig. 1f).

Taken together, these results showed that PB2 inhibited proliferation, induced cell cycle arrest in the S phase, and induced apoptosis of HCC cells.

### PB2 suppressed the growth of HCC in vivo

Because PB2 suppressed the proliferation of HCC cell lines in vitro, we also established a xenograft model using HCC-LM3 injection to verify whether PB2 inhibited the growth of HCC in vivo. As shown in Fig. 1g, oral intake of 100 mg/kg PB2 per day decreased the tumor volume significantly when compared to the NC group. H&E staining showed that there were many cancer cells and neo-vessels diffused in the solid neoplastic tissue in the NC group (Fig. 1h), while in the PB2 group, there were more lesions of necrosis and less neo-vessels. These results indicated that PB2 promoted necrosis in xenograft tumors to inhibit the growth of HCC. The TUNEL assay also showed that there were more apoptotic cells in the PB2 group than in the NC group (Fig. 1h), which indicated that PB2 induced apoptosis in the xenograft tumor in vivo. We also examined the pathological manifestations of the heart, kidney and lung. The results showed that there was no distant organ metastasis in both the NC and PB2 groups (Fig. 1i), and the H&E staining showed that the oral intake of PB2 (100 mg/kg daily) for 1 month did not harm these organs. Overall, these results suggested that PB2 promoted necrosis and induced apoptosis, to suppress the growth of HCC in vivo.

### PB2 reduced the aerobic glycolysis level in HCC cell lines

**Fig. 2:**
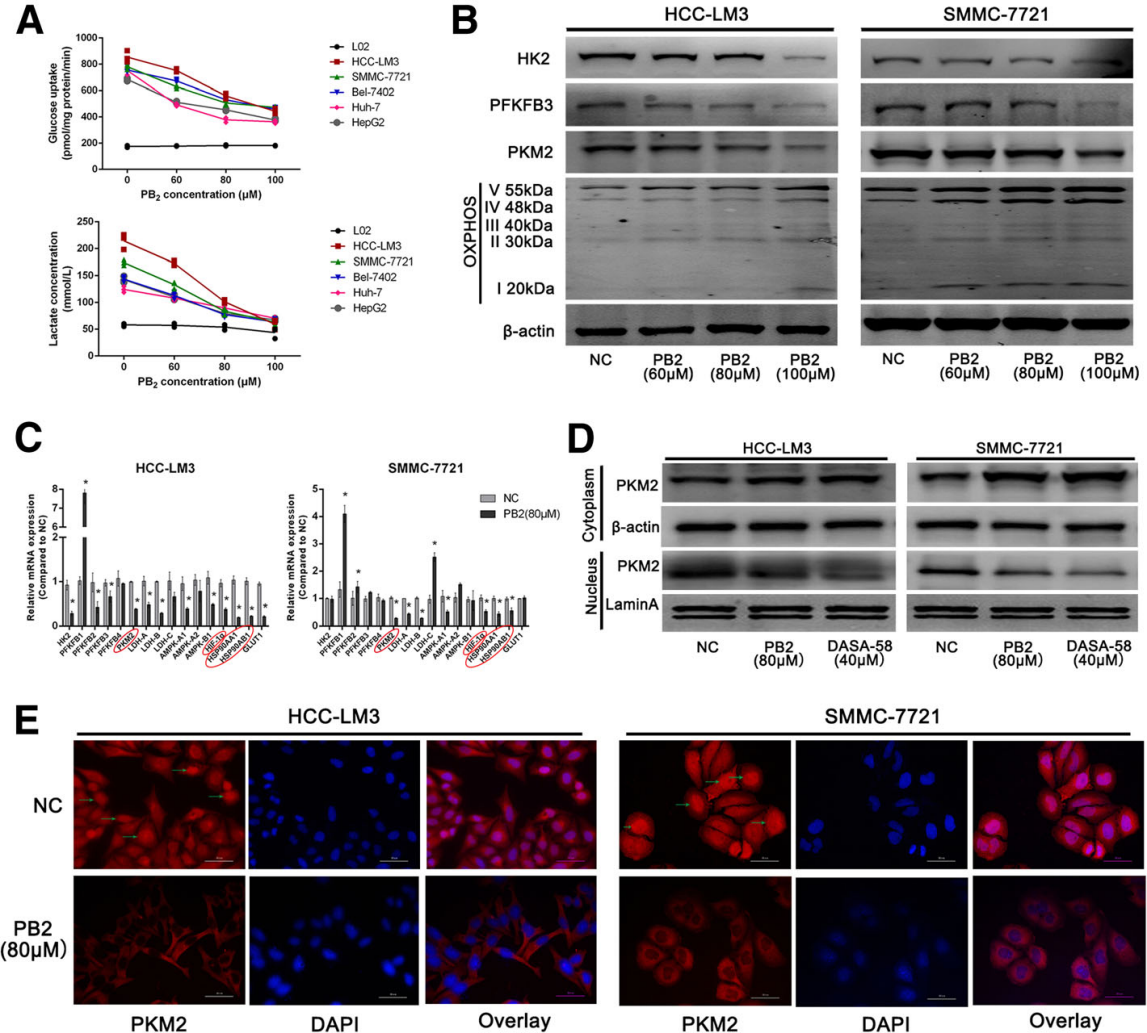
PB2 inhibited the aerobic glycolysis level in HCC cells. **a** The effects of PB2 on glucose uptake and lactate concentration in five HCC cell lines and L02 cells. **b** Western blotting analysis of three rate-limiting enzymes and OXPHOS. PB2 treatment decreased the expression of HK2, PFKFB3, and PKM2, while it increased OXPHOS expression in a dose-dependent manner. **c** The qPCR analysis of 16 aerobic glycolysis-related genes. *PKM2*, *HIF-1α*, and *HSP90* were the most changed genes after PB2 treatment. **d** A PKM2 agonist (DASA-58, 40 μM) was used to determine the role and location of PKM2 during the HCC inhibition effect of PB2 by western blotting. PB2 inhibited the expression of PKM2 in the nucleus. **e** The immunofluorescence staining of PKM2 in HCC cells (original magnification, 400×). PKM2 was detectable in both the cytoplasm and nucleus in the NC group, while PB2 not only suppressed the expression but also the nuclear localization of PKM2. ^*^P < 0.05 vs. the NC group (2C: one-way ANOVA)

Aerobic glycolysis is one of the most important hallmarks of cancer cells, including HCC cells. We therefore examined the effect of PB2 on aerobic glycolysis in HCC cells. As shown in Fig. 2a, all five of the HCC cell lines possessed a higher level of glucose uptake and supernatant lactate than the L02 cells, which confirmed an enhanced glycolysis level in HCC cells, and both the HCC-LM3 and SMMC-7721 cells were active in aerobic glycolysis. However, PB2 treatment for 24 h inhibited glucose uptake and supernatant lactate levels in a dose-dependent manner. The three rate-limiting enzymes of aerobic glycolysis, including HK2, PFKFB3 and PKM2, and the mitochondria OXPHOS enzyme expression levels were further detected by western blotting. The results showed that PB2 suppressed the expression of HK2, PFKFB3, and PKM2, while enhancing the expression of OXPHOS in both HCC-LM3 and SMMC-7721 cells (Fig. 2b). Together, these results showed that PB2 inhibited aerobic glycolysis and improved OXPHOS in HCC cell lines.

### PB2 downregulated the expression of PKM2 in HCC cells during the inhibition of aerobic glycolysis

We then explored the possible target of PB2 during the inhibition of aerobic glycolysis. The transcription levels of 16 aerobic glycolysis-related genes were detected by qPCR in both HCC-LM3 and SMMC-7721 cells (Fig. 2c). The results showed that eight genes could be regulated by PB2 consistently in both cells, including *PFKFB3, PKM2, LDH-A, LDH-B, AMPK-A1, HIF-1α, HSP90AA1,* and *HSP90AB1*. Because the protein levels of PFKFB3 were opposite to those of the qPCR results, we chose PKM2 as the most important protein (the reason was shown in Additional file 1: Table S1 and Additional file 1: Figure S1). Since PKM2 can translocate into nucleus, to determine the role of PKM2 during the inhibitory effect of PB2, a PKM2 activator DASA-58 (40 μM) was tested [15, 25]. After treatment with DASA-58 or PKM2, the protein levels of PKM2 in the nucleus were downregulated in both HCC-LM3 and SMMC-7721 cells (Fig. 2d), while the PKM2 levels in the cytoplasm were upregulated. These results indicated that PB2 can inhibit the nuclear translocation of PKM2 as DASA-58. IF staining results were also similar to Fig. 2d, which showed that there was higher PKM2 expression located in both the cytoplasm and nucleus of NC HCC cells (Fig. 2e); However, PB2 treatment could not only reduce the fluorescence intensity of PKM2, but it could also inhibit the expression of PKM2 in the nucleus, which might be important for the inhibitory effects of PB2. These results preliminarily suggested that PB2 targeted PKM2 and inhibited both the expression and nuclear translocation of PKM2, which is one of the rate-limiting enzymes during glycolysis, to inhibit the growth of HCC in vitro.

### PKM2 was the target of PB2, and its overexpression or knockdown influenced the effect of PB2 in HCC cell lines

**Fig. 3:**
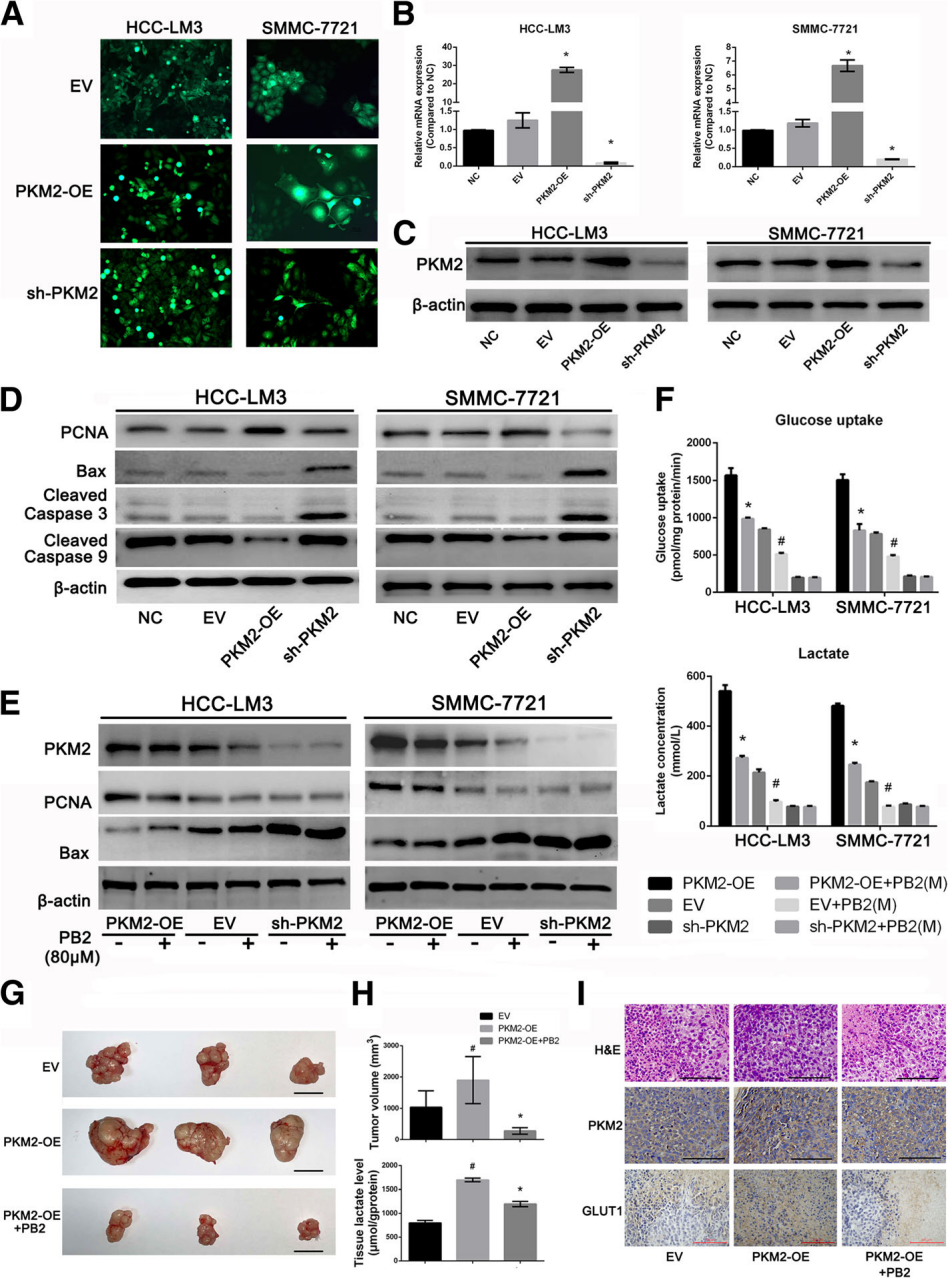
PKM2 is the target of PB2, and the overexpression or knockdown of PKM2 influenced the effect of PB2 in HCC cell lines. **a** The fluorescence of HCC cells after transfection with PKM2-OE or sh-PKM2 lentiviruses (original magnification, 200×). **b**, **C** The transfection efficiency was verified by qPCR and western blotting. ^*^P < 0.05 vs. the NC group. **d** The effects of PKM2 overexpression or knockdown on proliferation- and apoptosis-related markers, by western blotting. **e** The effects of PB2 on the proliferation and apoptosis in PKM2-OE or sh-PKM2 HCC cells. **f** The effects of PB2 on the glucose uptake and lactate levels in PKM2-OE or sh-PKM2 HCC cells. **g** The gross images of tumors in EV, PKM2-OE, or PKM2-OE + PB2 groups (bars: 10 mm). **h** The tumor volumes and tumor tissue lactate levels in EV, PKM2-OE, or PKM2-OE + PB2 groups. **i** The H&E staining and IHC staining of PKM2, GLUT1 (bars: 100 μm). ^*^P < 0.05 vs. the PKM2-OE group; # *P* < 0.05 vs. the EV group (3**b**: one-way ANOVA; 3**f** & 3**h**: Student’s t-test)

Lentiviruses, which contained PKM2-OE, sh-PKM2, or an empty vector were transfected into both HCC-LM3 and SMMC-7721 cells. The transfection efficiency was verified by green fluorescent protein imaging, qPCR, and western blotting, which suggested successful transfections (Fig. 3a, b, and c). The roles of PKM2 in the proliferation and apoptosis of HCC cell lines were then determined. As shown in Fig. 3d, the protein levels of PCNA were enhanced in PKM2-OE groups and reduced in the sh-PKM2 groups, while the levels of Bax, cleaved caspase 3, and cleaved caspase 9 were decreased in the PKM2-OE groups and increased in the sh-PKM2 groups. The expression of these proteins did not significantly change in the EV groups. Furthermore, PB2 was also administered to both of the transfected HCC cell lines. The western blotting results showed that the expression of PKM2 could still be downregulated by PB2 in PKM2-OE, EV, and sh-PKM2 HCC cells (Fig. 3e). Moreover, in PKM2-OE HCC cells, PB2 treatment decreased the expression of PCNA and increased the expression of Bax. However, in sh-PKM2 HCC cells, PB2 treatment did not influence the expression of both PCNA and Bax. To examine whether PB2-reduced glucose uptake and lactate levels were dependent on PKM2, we further examined the glucose uptake and lactate levels after PB2 treatment, and the results were similar to the western blotting results (Fig. 3f). These results further indicated that after the knockdown of PKM2, PB2 could not suppress the proliferation, induce apoptosis or inhibit glycolysis levels of HCC cells, which meant that PKM2 was actually the target of PB2. Furtherly, the results in Fig. 3g & h showed that PKM2-OE group displayed larger tumor volumes and higher lactate levels than the EV group, while PB2 treatment could inhibited the tumor volumes and tissue lactate levels effectively. The H&E staining and IHC staining of PKM2 and GLUT1 also showed there were more cancer cells and neo-vessels, and higher level of PKM2 and GLUT1 (which represented for the glucose uptake levels) expression in the PKM2-OE group, while in the PB2-treated group, the situation was improved (Fig. 3i). These indicated that overexpression of PKM2 would promote the formation and volume of tumor tissues in vivo by enhancing aerobic glycolysis, while PB2 treatment, which was proved to targeted on PKM2, was effective to reduce the malignancy of tumors. Together, these results showed that PKM2 promoted the proliferation and ameliorated apoptosis of HCC cells, and that PKM2 was actually the target of PB2. By targeting PKM2, PB2 inhibited the growth of HCC in vitro and in vivo.

### PB2 disrupted the interaction between PKM2/HSP90/HIF-1α and inhibited the nuclear translocation of PKM2

**Fig. 4:**
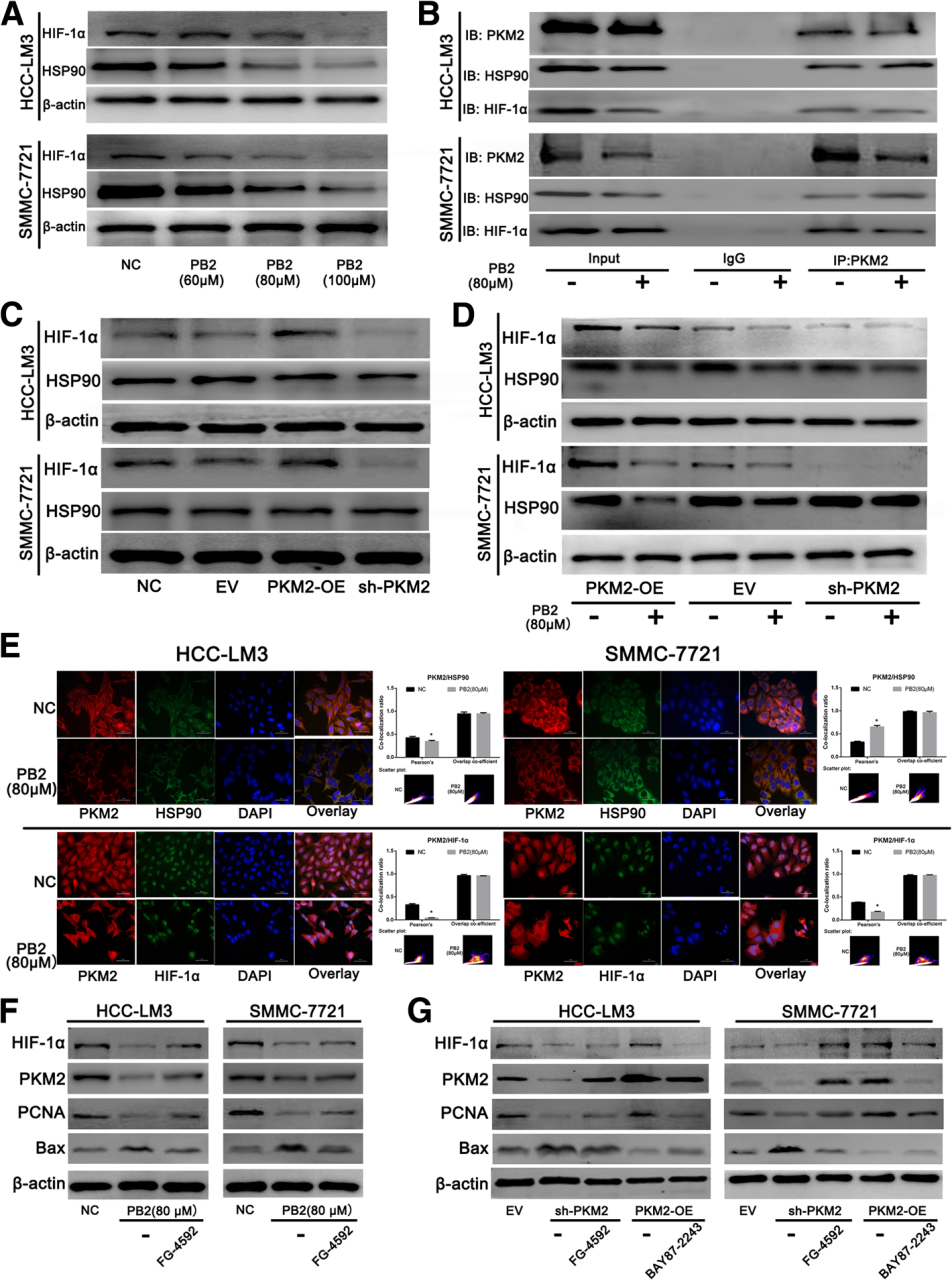
PB2 disrupted the interaction between PKM2/HSP90/HIF-1α in HCC cells. **a** Western blotting showed that PB2 downregulated the expression of HIF-1α and HSP90 in HCC cells. **b** The co-immunoprecipitation assay. Both HIF-1α and HSP90 were pulled-down by PKM2. The levels of the PKM2/HIF-1α complex in PB2 groups were lower than those in the normal control (NC) groups, while the levels of PKM2/HSP90 complex in the PB2 groups were higher than those in the NC groups. **c** The effects of PKM2 overexpression or knockdown on the expression of HIF-1α and HSP90, by western blotting. **d** PB2 reduced the expression of HIF-1α and HSP90 in PKM2-OE or sh-PKM2 HCC cells when compared to the EV group. **e** Double-immunofluorescence (IF) staining of HCC cells (original magnification, 400×). PKM2 was stained as red, HIF-1α or HSP90 was stained as green. HSP90 was mainly localized in the cytoplasm of HCC cells, while HIF-1α was localized in the nucleus. The double-IF staining and co-localization suggested that PB2 promoted the co-localization of PKM2/HSP90 in the cytoplasm, while it suppressed the co-localization of PKM2/HIF-1α in the nucleus, which was quantified using Pearson’s co-localization coefficient. **f** Effects of HIF-1α activator FG-4592 on PB2-treated HCC cells. **g** Effects of HIF-1α activator FG-4592 and HIF-1α inhibitor BAY87–2243 on PKM2-overexpression or PKM2-knockout HCC cells. ^*^P < 0.05 vs. the NC group (4**e**: Student’s t-test)

PKM2 can also serve as a transcriptional cofactor to promote gene transcription in cancer cells by translocating into the nucleus [16, 26]. The results in Fig. 2d & e showed that PB2 could inhibited the nuclear translocation of PKM2. Figure 2c shows that *HSP90* and *HIF-1α* were two genes that significantly changed after PB2 treatment. The protein expression levels of these proteins were therefore determined using western blotting. The results showed that PB2 reduced the expressions of both HSP90 and HIF-1α in a dose-dependent manner in HCC cells, which was consistent with the qPCR results (Fig. 4a). It has been reported that nuclear PKM2 may directly interact with HIF-1α by forming a complex with it to promote the transcription of HIF-1α target genes [14, 15]. Moreover, the cytoplasmic HSP90 has also been found to interact with PKM2 to ensure the stability of PKM2 [27]. A co-IP assay was then conducted to determine the relationship between PKM2, HSP90, and HIF-1α in HCC cell lines (Fig. 4b). The results showed that both HIF-1α and HSP90 were pulled-down by PKM2. The level of the PKM2/HIF-1α complex in the PB2 groups was lower than that in the NC groups, while the level of the PKM2/HSP90 complex in the PB2 groups was higher than that in the NC groups. We then detected the expression of HIF-1α and HSP90 during the changes of PKM2 levels. The western blotting results showed that overexpression of PKM2 in both HCC cell lines promoted the expression of HIF-1α, and knockdown of PKM2 decreased the HIF-1α levels (Fig. 4c), while the changes of PKM2 had no effect on the expression of HSP90, which was probably because HSP90 was not the target gene of PKM2 or HIF-1α (PB2 may influence the levels of HSP90 through unknown mechanisms in the study, for example, PB2 may influence the stability or the degradation of HSP90). These effects on HIF-1α were also reduced by PB2 treatment of PKM2-OE HCC cells (Fig. 4d). To furtherly verify the relationship among these proteins, double-IF was conducted (Fig. 4e). The results showed that PB2 decreased the expression of both HIF-1α and HSP90 in the two cell lines, which was consistent with the western blotting results. Moreover, PB2 promoted co-localization of PKM2/HSP90 in the cytoplasm, while it suppressed the co-localization of PKM2/HIF-1α in the nucleus.

To furtherly explore the relationship between PKM2 and HIF-1α, we firstly detected the involvement of NF-κB and Stat3 during the study. The results in Additional file 1: Figure S4 showed that the protein levels of Stat3, p-Stat3(Tyr705) and NF-κB were not changed after PB2 treatment in both HCC cell lines. These results indicated that both Stat3 and NF-κB were not involved in the effect of PB2 on HCC. Secondly, to verify whether the downregulation of PKM2 by PB was through HIF-1α-mediated transcriptional regulation suppression or not, a HIF-1α activator (FG-4592, 50 μM) and a HIF-1α inhibitor (BAY87–2243, 10 μM) were used. As Fig. 4f showed, the protein levels of HIF-1α, PKM2, PCNA and Bax were detected after tread with PB2 and/or HIF-1α activator FG-4592. The results revealed that the effect of PB2 could be improved by FG-4592 treatment, which indicated that the effects of PB2 on HCC cells may through the inhibition of HIF-1α. Since we have proved that PKM2 was the target during the effect of PB2 on HCC in Fig. 3, we furtherly verify the relationship between PKM2 and HIF-1α in PKM2-OE or sh-PKM2 groups. In Fig. 4g, in sh-PKM2 groups, the levels of HIF-1α, PKM2 and PCNA were lower than that in EV groups, and Bax levels were higher. However, after treated with HIF-1α activator FG-4592, the protein levels of them were recuperative. Moreover, in PKM2-OE groups, the levels of these protein can also be reversed by HIF-1α inhibitor BAY87–2243. These results meant that HIF-1α was the downstream regulator of PKM2. In other word, we proved that the downregulation of PKM2 by PB was directly through HIF-1α-mediated transcriptional regulation suppression.

Together, these results suggested that PB2 also inhibited the expression of HIF-1α and HSP90, and furtherly disrupted the interaction between PKM2/HSP90/HIF-1α and inhibited the nuclear translocation of PKM2, resulting in an inhibition in HIF-1α-mediated transcription.

### PB2 enhanced the chemosensitivity of SORA on HCC, both in vivo and in vitro

**Fig. 5:**
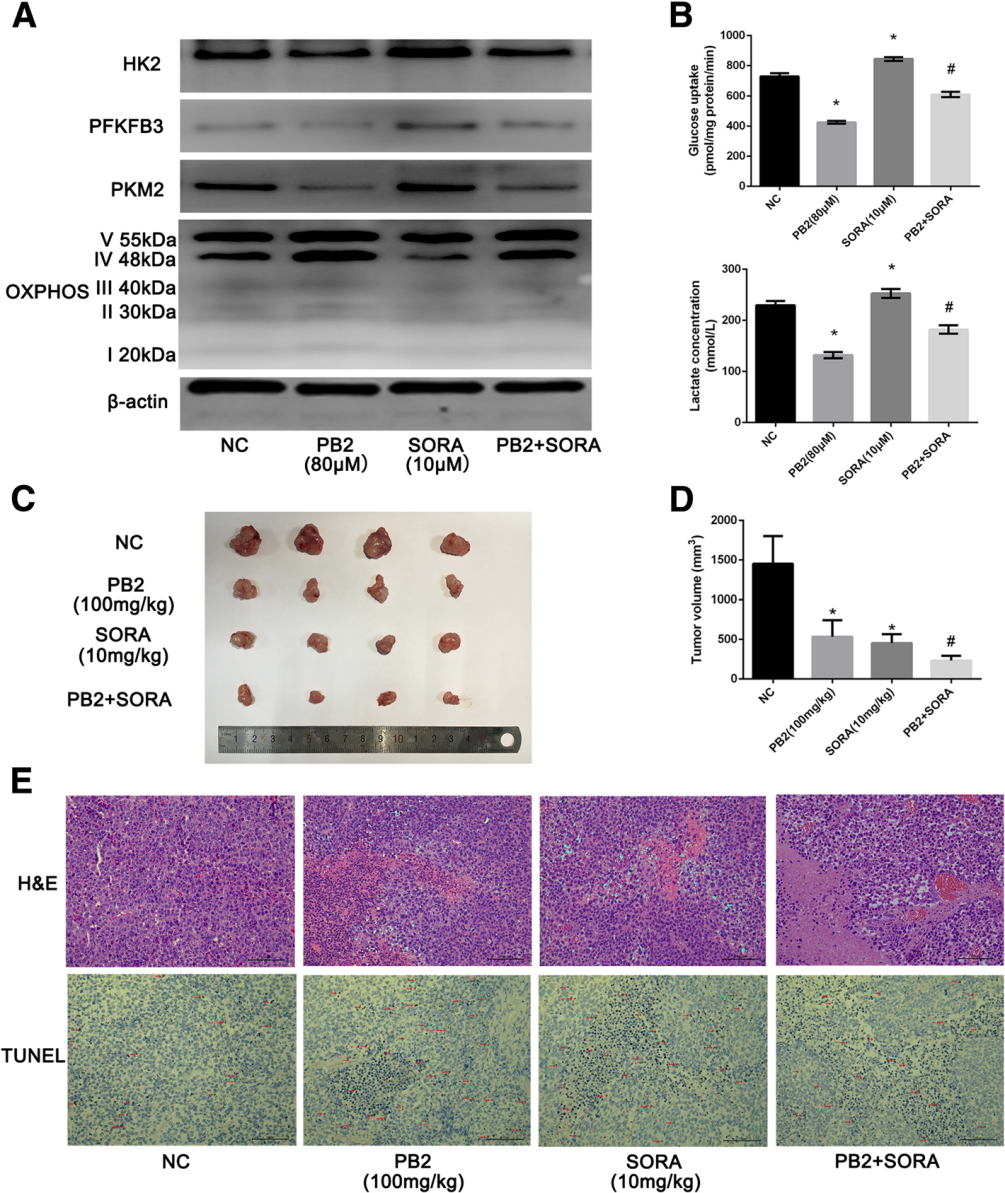
PB2 enhanced the chemosensitivity of sorafenib (SORA) in HCC cells in vivo and in vitro. **a** The combined effect of PB2 and SORA on the metabolic enzymes of HCC cells, by western blotting analysis. **b** The combined effect of PB2 and SORA on the glucose uptake and supernatant lactate levels in HCC cells. **c**, **d** PB2 and SORA co-treatment suppressed the growth and volume of mouse xenograft tumors. **e** The hematoxylin and eosin TUNEL staining of tumor sections. PB2 and SORA co-treatment increased the necrosis and apoptosis in tumor tissues. ^*^P < 0.05 vs. the normal control group; ^#^*P* < 0.05 vs. the SORA group (5B&5D: one-way ANOVA)

SORA is an ideal targeted drug to treat HCC [28]. However, there has been increasing drug resistance because it has been reported that SORA enhances aerobic glycolysis and inhibits OXPHOS in HCC cells [29, 30]. Because we have shown that PB2 was an effective treatment for growth inhibition and glycolysis both in vivo and in vitro, the combination treatment of SORA and PB2 might show a synergic effect on HCC. As shown in Fig. 5a, western blotting analysis of these rate-limiting enzymes showed that 10 μM SORA treatment reduced the OXPHOS expression in HCC-LM3 cells, while it increased the levels of HK2, PFKFB3, and PKM2, which has been reported to be responsible for the SORA resistance [29]. However, if combined with PB2, these effects were reversed. The glucose uptake assay and determination of supernatant lactate levels was the same as the western blotting results (Fig. 5b). The xenograft model showed that both PB2 and SORA were effective in decreasing the xenograft tumor volumes, but the combination treatment of PB2 and SORA revealed a stronger effect (Fig. 5c and d). In addition, H&E staining and TUNEL staining showed that the combination treatment of PB2 and SORA induced more necrosis and apoptosis of tumor cells when compared with treatment with PB2 or SORA alone (Fig. 5e). Overall, these results suggested that PB2 synergically enhanced the effect of SORA on HCC both in vivo and in vitro.

## Discussion

HCC is considered to be an aggressive tumor because of its frequent tumor recurrences and metastases [2, 6]. It is often diagnosed at an advanced stage with a poor prognosis and high mortality. There are six hallmarks contributing to the malignancy of HCC, including sustaining proliferation, growth inhibition evading, immortality, angiogenesis, invasion and metastasis, and the Warburg effect [8, 31, 32]. The enhancement of aerobic glycolysis in HCC has been widely reported, because aerobic glycolysis can provide a more efficient energy and metabolic substrate supply for the tumor’s high proliferation, and the production of lactate will result in an acid environment to facilitate the metastasis and avoid immune surveillance of cancer cells [31, 32]. Our study determined the aerobic glycolysis levels in five HCC cell lines and the LO2 normal liver cell line. The results showed that all five HCC cell lines, especially HCC-LM3 and SMMC-7721 cells, possessed an increased level of glycolysis when compared with the LO2 cells, which was consistent with previous studies. Based on the metabolic characteristics of HCC, a better investigation of drugs targeted to aerobic glycolysis is a new therapeutic strategy against HCC.

PB2 is a type of flavonoid known for its anti-oxidant bioactivity [17]. Many studies have reported that proanthocyanidins also possess anti-tumor properties, including the inhibition of breast cancer, prostate cancer, lung cancer, and colorectal cancer [24, 33, 34]. Recent studies have also reported that grape seed proanthocyanidins inhibit proliferation of HepG2 cells or the growth of liver cancer in a xenograft model by inhibiting apoptosis and angiogenesis [23, 33]. In our study, we found that PB2 inhibited proliferation and induced cell cycle arrest and apoptosis of HCC cells, as well as inhibiting the growth in a mouse xenograft model without apparent toxicity to the main organs.

Because aerobic glycolysis is important in the growth HCC, and there are few studies about the effect of PB2 on the aerobic glycolysis of HCC, we characterized the effects of PB2 on aerobic glycolysis of HCC. Our results showed that PB2 reduced aerobic glycolysis by suppressing glucose uptake and supernatant lactate levels, and enhanced OXPHOS in HCC cell lines. The detailed mechanism of PB2 effects was further investigated. After screening a series of genes, we found that *PKM2, HSP90*, and *HIF-1α* were the most obviously altered genes following PB2 treatment. Moreover, the effect of PB2 was similar to DASA-58 co-treatment, which confirmed that PKM2 was the target of PB2.

**Fig. 6:**
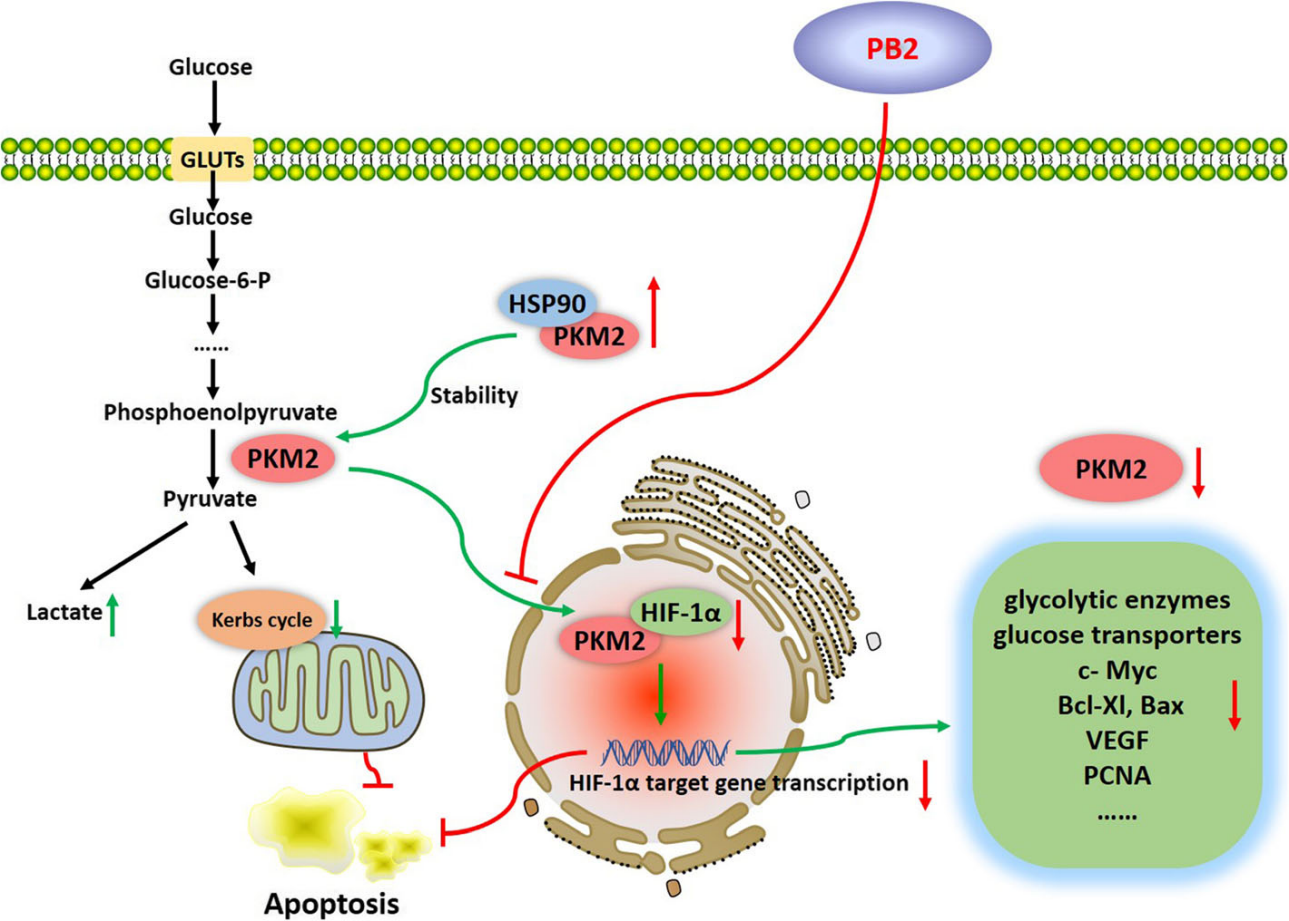
The mechanism of the PB2 effect on glycolysis inhibition in HCC cells. PKM2 is the last rate-limiting enzyme during glycolysis. The cytoplasmic HSP90 bound PKM2 to maintain the stability of PKM2. While PKM2 also translocated into the nucleus and interacted with HIF-1α to promote the transcription of HIF-1α targeted genes, such as the key glycolytic enzymes (including PKM2), the glucose and lactate transporters, c-Myc, Bcl-xL, and VEGF. In our study, we found that PB2 inhibited the expression and nuclear translocation of PKM2, therefore it disrupted the interaction between PKM2/HSP90/HIF-1α, to suppress aerobic glycolysis, proliferation, and to trigger apoptosis via HIF-1α-mediated transcription suppression in HCC

PKM2 is a rate-limiting enzyme during glycolysis. Overexpression of PKM2 has been widely recognized in HCC, which is related to the metastasis and poor prognosis of HCC and drug resistant to SORA [16, 35, 36]. The role of PKM2 in HCC is complicated. PKM2 located in the cytoplasm catalyzes the last step of glycolysis by transferring phosphoenolpyruvate into pyruvate, therefore resulting in the promotion of glycolysis, a faster energy supply, and the increased production of metabolic intermediates [36]. However, PKM2 also translocates into the nucleus and forms a complex with HIF-1α, where it acts as a transcriptional coactivator of HIF-1α to promote the transcription of HIF-1α targeted genes, such as the key glycolytic enzymes (including *PKM2*), the glucose and lactate transporters, *c-Myc, Bcl-xL*, and *VEGF* [14, 15]. Moreover, our study showed that the overexpression of PKM2 promoted the proliferation and inhibited apoptosis of HCC cells through the regulation of related gene expressions (Fig. 6). Hence, the inhibition of PKM2 was critical for the suppression effect of PB2 on HCC.

HIF-1α is an activated transcription factor, which responds to hypoxic stress during the development of HCC [37]. HSP90 has been found to be a potential biomarker for the diagnosis of HCC, which is a type of conserved molecular chaperone facilitating protein folding, protein complex assembly, and protein degradation [38, 39]. We focused on the relationship between PKM2, HSP90, and HIF-1α, which were significantly reduced by PB2 treatment in parallel with PKM2. Firstly, the overexpression or knockdown of PKM2 showed that PKM2 inhibited the expression of HIF-1α, but had no influence on the expression of HSP90, which indicated that *HIF-1α*, but not *HSP90,* was probably the target gene of PKM2. Secondly, except for the inhibition of PKM2 expression, PB2 treatment decreased the nuclear translocation of PKM2. HSP90, which has been found to be overexpressed in HCC as well, plays a critical role in regulating the proliferation, apoptosis, and metastasis of tumor cells [40–42]. HSP90 can act as a molecular chaperone of PKM2 in the cytoplasm to bind with PKM2, to ensure the stability of the PKM2 protein [27]. The Co-IP assay and double-IF results suggested that PB2 promoted the co-localization of PKM2/HSP90 in the cytoplasm, while it suppressed the co-localization of PKM2/HIF-1α in the nucleus. Thirdly, as previously mentioned, PKM2 may translocate to the nucleus and bind with HIF-1α to cause an energy metabolism shift from OXPHOS to glycolysis, as well as the promotion of metastasis, angiogenesis, and radiation resistance, by stimulating the transcription of HIF-1α target genes, including PKM2 [14, 15, 31]. The results in Fig. 4f & g also demonstrated that HIF-1α was the downstream regulator of PKM2. Based on these possibilities, we suggest that PB2 inhibits the expression of PKM2, HIF-1α and HSP90, and further disrupts the interaction between PKM2/HSP90/HIF-1α to inhibit the nuclear translocation of PKM2, which finally leads to the inhibition of aerobic glycolysis in HCC via HIF-1α-mediated transcription suppression.

SORA is a first-line treatment of advanced HCC, which can increase the survival of HCC patients [43]. However, the resistance to SORA develops in long-term use, varying from months to years, which is because of various mechanisms [44]. Pan et al. found that PKM2 contributed to the drug resistance to SORA through the increase of glycolysis flux [45]. HIF-1α contributes to SORA resistance by stabilizing and increasing the expression of the multidrug resistance protein 1, the glucose transporter 1, and vascular endothelial growth factor [46]. In addition, Augello et al. reported that a HSP90 inhibitor synergistically increased the effect of SORA on HCC [47]. In our study, we found that PB2 enhanced the effect of SORA on HCC both in vivo and in vitro by targeting PKM2. Emerging evidence has revealed that botanic flavonoids, including oroxylin A, tannic acid, and genistein, enhance the chemosensitivity to SORA [11, 48, 49]. Because PB2 is a nontoxic agent to L02 cells and mice, PB2 is a promising agent to act as a chemopreventive or chemotherapeutic medicine to treat HCC.

## Conclusions

In conclusion, our study suggested an effective and safe agent for the treatment of HCC. PB2 inhibited the expression and nuclear translocation of PKM2, to disrupt the interaction between PKM2/HSP90/HIF-1α, subsequently suppressing aerobic glycolysis, proliferation, and triggering apoptosis in HCC cells via HIF-1α-mediated transcription suppression.

## Additional file

Additional file 1: Supplementary Materials (DOCX 833 kb)

## Abbreviations

BCA: bicinchoninic acid
CCK-8: cell counting kit
Co-IP: co-immunoprecipitation
DMSO: dimethyl sulfoxide
EV: empty vector
FBS: fetal bovine serum
H&E: hematoxylin and eosin
HBV/HCV: hepatitis B/C viruses
HCC: hepatocellular carcinoma
HIF-1α: hypoxia-inducible factor 1α
HK: hexokinase
HSP90: heat shock protein 90
IF: immunofluorescence
IHC: immunohistochemistry
NC: normal control
OXPHOS: oxidative phosphorylation
PB2: proanthocyanidin B2
PBS: phosphate-buffered saline
PCNA: proliferating cell nuclear antigen
PFK: fructose-6-phosphokinase
PI: propidium iodide
PKM2: pyruvate kinase M2
qPCR: quantitative real-time polymerase chain reaction
RIPA: radioimmunoprecipitation assay
RT-PCR: reverse transcription-polymerase chain reaction
SORA: sorafenib
TUNEL: terminal deoxynucleotidyl transferase dUTP nick end labeling
