# Supplementary material for: PKM2 is the target of proanthocyanidin B2 during the inhibition of hepatocellular carcinoma
Source: J Exp Clin Cancer Res. 2019 May 17;38:204. doi: 10.1186/s13046-019-1194-z (PMC6525465; doi:10.1186/s13046-019-1194-z)
Supplement: Supplementary file 1 — Supplementary Materials (DOCX 833 kb) [file 13046_2019_1194_MOESM1_ESM.docx]

**Supplementary Materials**

**1. Table S1**

**Table S1 The evaluation of some significant genes.**

| Gene | HCC-LM3 | | |  | SMMC-7721 | | |
| --- | --- | --- | --- | --- | --- | --- | --- |
|  | NC* | PB2* | Fold^#^ |  | NC* | PB2* | Fold^#^ |
| *PKM2* | 0.99 | 0.39 | 2.57 |  | 1.03 | 0.29 | 3.53 |
| *LDH-A* | 1.01 | 0.48 | 2.11 |  | 1.01 | 0.48 | 2.11 |
| *LDH-B* | 1.00 | 0.29 | 3.40 |  | 1.02 | 0.29 | 3.49 |
| *AMPK-A1* | 0.95 | 0.39 | 2.42 |  | 1.08 | 0.53 | 2.04 |
| *HIF-1A* | 0.96 | 0.38 | 2.52 |  | 1.02 | 0.54 | 1.89 |
| *HSP90AA1* | 1.03 | 0.20 | 5.23 |  | 1.00 | 0.45 | 2.23 |
| *HSP90AB1* | 1.01 | 0.22 | 4.49 |  | 0.98 | 0.57 | 1.73 |

*The mean value of qPCR results in Figure 2C.

#Fold = mean (NC group) / mean (PB2group).

**
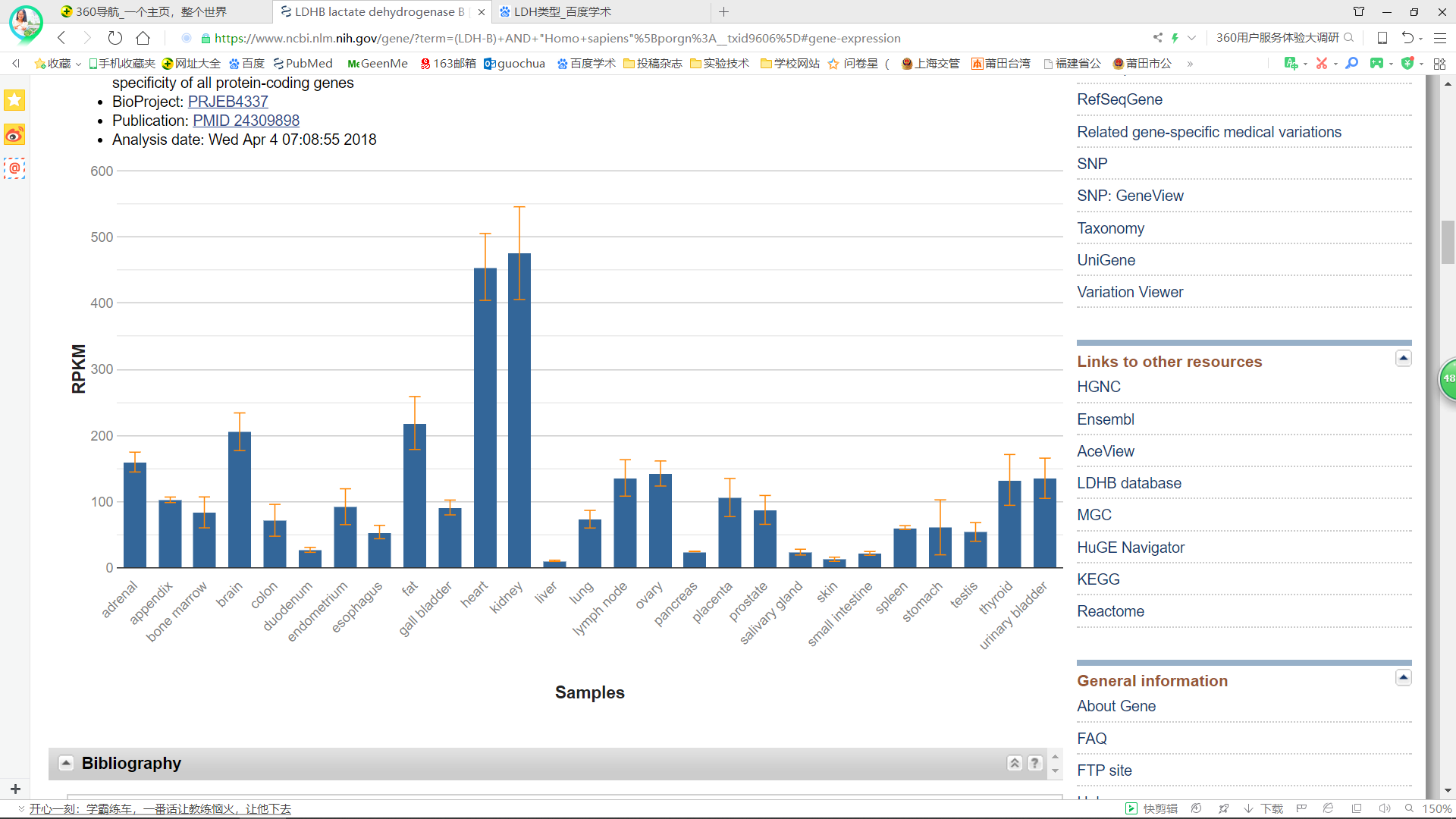
2. Figure S1**

**Figure S1: The expression levels of *LDH-B* mRNA in different tissues.** The expression of *LDH-B* is low in liver. (The image was downloaded from GenBank: <https://www.ncbi.nlm.nih.gov/gene/3945#gene-expression>)


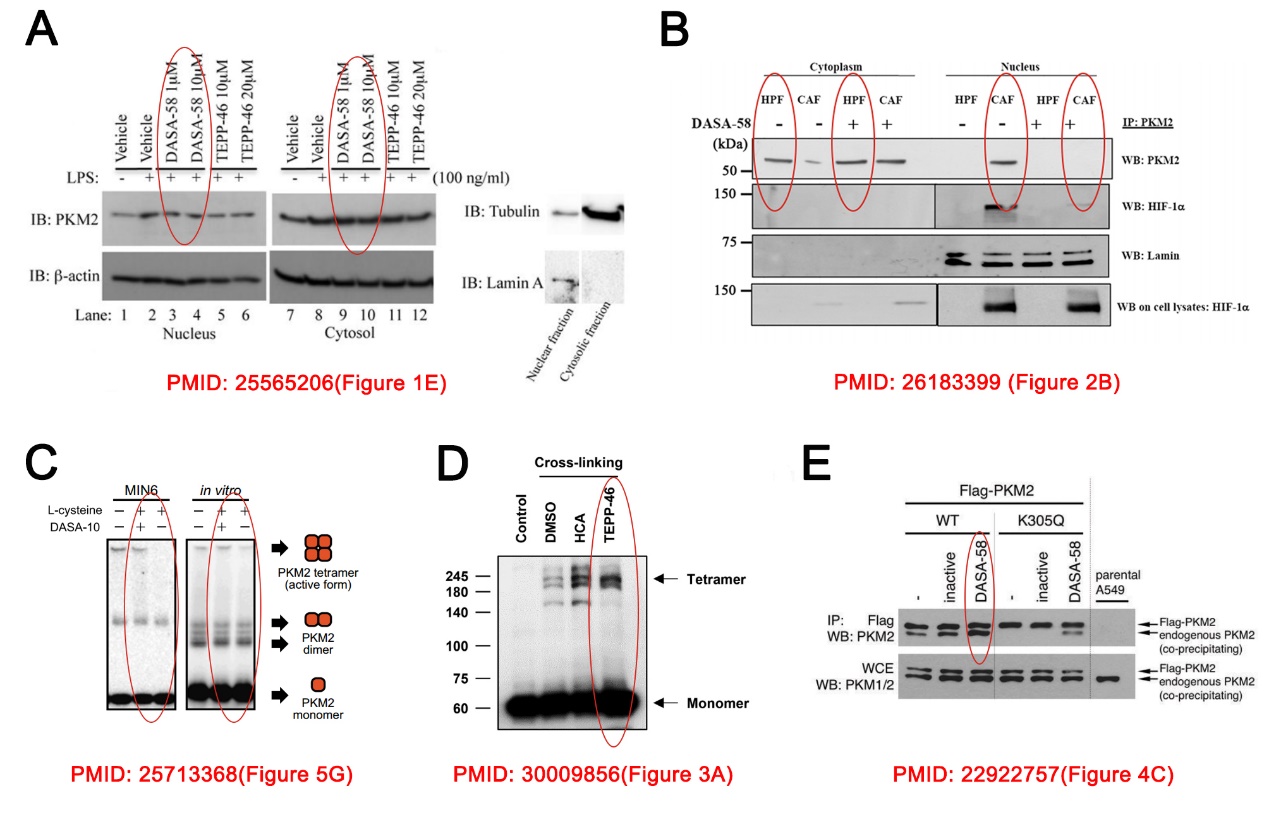
**3. Figure S2**

**Figure S2：The effect of PKM2 activators on PKM2 protein level.** (A, B) DASA-58 treatment can increase PKM2 levels in the cytoplasm and decrease PKM2 levels in the nucleus. (C) DASA-10 increased the PKM2 tetramer level, while the PKM2 monomer level didn’t change. (D) TEPP-46 increased both the PKM2 tetramer and PKM2 monomer levels. (E) DASA-58 can promote the interaction between exogenous PKM2 and endogenous PKM2.


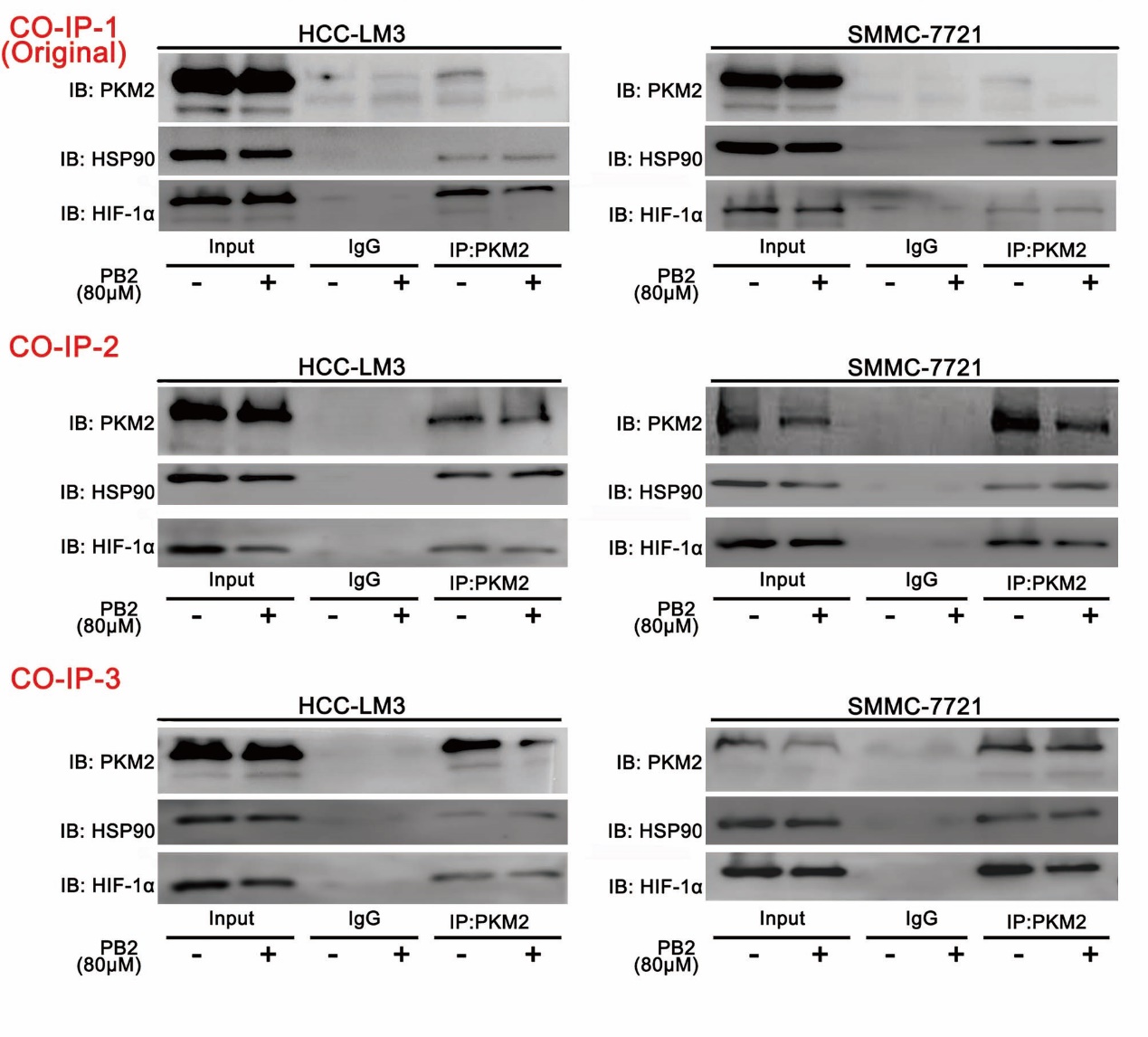
**4. Figure S3**

**Figure S3: The three independent co-IP assay results during the study.** CO-IP-1 were the 1^st^ and original images for Figure 4B, which were not ideal so that made readers confused. While the 2^nd^ and the 3^rd^ results were better than the 1^st^ one. Therefore, the 2^nd^ results were used for layout in revised Figure 4B.

**
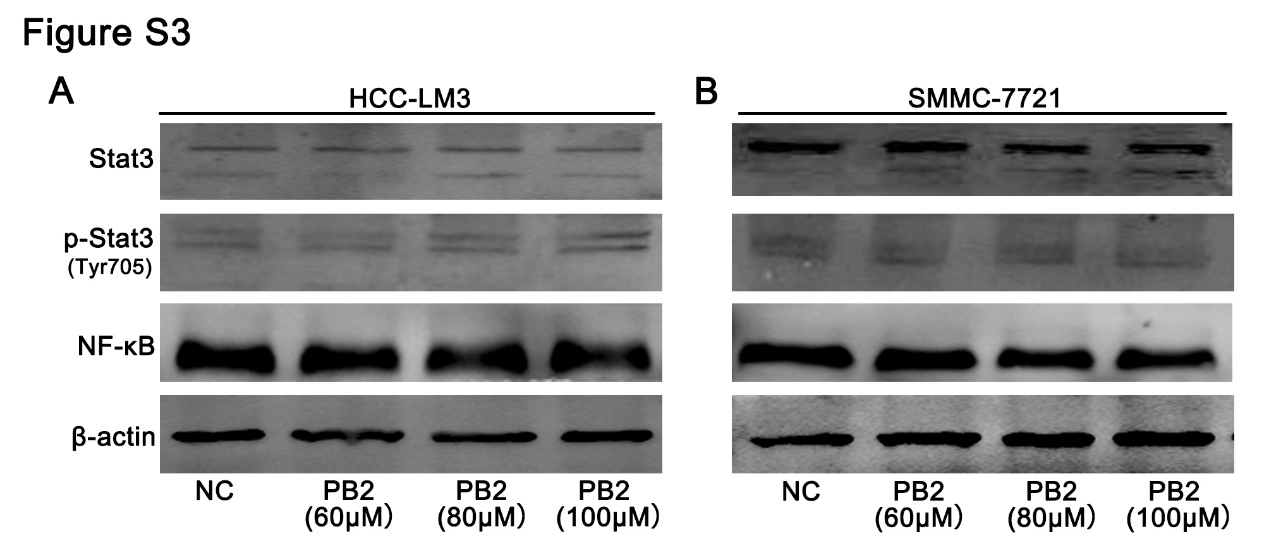
5. Figure S4**

**Figure S4: Effects of Stat3 and NF-κB on HIF-1α expression by PB2 treatment.** (A, B) Both HCC-LM3 and SMMC-7721 cells were treated with 60, 80 or 100 μM PB2, and the protein levels of Stat3, p-Stat3(Tyr705) and NF-κB were detected.
